# Gynecologic radiation oncology practice: retrospective analysis of a tertiary radiotherapy referral center

**DOI:** 10.3389/fonc.2026.1786400

**Published:** 2026-02-26

**Authors:** Alparslan Serarslan, Mert Büyükulaş, Bilge Gürsel, Nilgün Özbek Okumuş, Deniz Meydan

**Affiliations:** Department of Radiation Oncology, Faculty of Medicine, Ondokuz Mayıs University, Samsun, Türkiye

**Keywords:** brachytherapy, cervical cancer, endometrial carcinoma, gynecologic oncology, radiation oncology, radiotherapy

## Abstract

**Background:**

This study aimed to characterize gynecologic cancers in radiation oncology practice and compare survival outcomes with those reported in countries exhibiting a high or very high human development index.

**Methods:**

A retrospective review was conducted involving the medical records of all patients (n = 11,720) treated at our radiotherapy center between January 2012 and December 2022. Of these, 911 women with gynecologic cancers who underwent treatment and follow-up at our center were included in the analysis. Demographic, disease-related, and treatment-related characteristics, as well as relapse patterns and survival outcomes, were recorded and analyzed.

**Results:**

Gynecologic cancers (30%) ranked second among malignancies of women in radiation oncology, after breast cancer (35%). The two most common gynecologic cancers among all female malignancies in radiation oncology were corpus uteri cancer (20%, rank second after breast cancer) and cervical cancer (9%, rank third). With the exception of ovarian cancer, most gynecologic cancers were referred for radiotherapy at FIGO stages I–II (75%, n = 680). Curative and adjuvant radiotherapy rates were 95% (n = 771) and 75% (n = 580), respectively. The most frequently administered radiotherapy technique was external beam radiotherapy combined with brachytherapy (53%, n = 419). The rate of brachytherapy application in gynecologic radiation oncology was 82%. Palliative radiotherapy was most commonly administered for ovarian cancer (57%) in gynecologic oncology. The primary indication for palliative RT was pain management (42%). Relapse predominantly occurred as distant metastases in corpus uteri and cervical cancers, whereas other gynecologic cancers commonly exhibited locoregional disease. Survival outcomes, except for vaginal cancer, were comparable to those reported in the literature.

**Conclusions:**

Gynecologic cancers constitute a substantial proportion of radiation oncology practice in women. Survival outcomes at our center largely align with those observed in countries exhibiting a high or very high human development index.

## Introduction

Gynecologic cancers include uterine, cervical, ovarian, vulvar, vaginal, and fallopian tube malignancies, classified based on tumor location. These cancers represent key causes of morbidity and mortality worldwide (1). The incidence and survival rates of gynecologic cancers, whether considered collectively or individually, vary according to geographic region and a country’s level of development (2). Türkiye, an upper-middle-income country in the Europe and Central Asia region, has a very high human development index (HDI) (3). Studies of gynecologic cancers in Türkiye have indicated that survival rates are comparable to those in developed countries (4, 5).

The gynecologic oncology tumor board plays a critical role in the management of gynecologic cancers; radiation oncology is one of its key disciplines (6). There is evidence that timely treatment at fully equipped oncology centers, where multidisciplinary treatment plans are implemented, improves prognosis and reduces mortality associated with gynecologic cancers (4). Our university hospital, a tertiary cancer referral center, provides multidisciplinary care for gynecologic cancer patients from the Black Sea Region of Türkiye.

Considerable advancements have been made in gynecologic radiation oncology, particularly in radiotherapy (RT) planning and delivery techniques for both external beam radiotherapy (EBRT) and brachytherapy (BRA). RT can be administered at all stages of disease, including curative treatment for localized, regional, and oligometastatic cases, as well as palliative treatment for polymetastatic disease. Therefore, RT remains an integral component of multimodal treatment approaches for gynecologic cancers (1).

Although institutional reports on individual gynecologic cancers exist, there is a scarcity of studies that collectively analyze all gynecologic malignancies from a radiation oncology perspective, especially in settings with high brachytherapy utilization. This study aims to fill that gap by providing a holistic view of patient profiles, treatment patterns, and outcomes across all gynecologic cancer types treated in a tertiary radiotherapy center. To address this gap, a retrospective analysis was conducted involving gynecologic cancer patients who presented to our radiation oncology department from the Black Sea Region of Türkiye. This study is expected to serve as a valuable reference for Türkiye, the surrounding region, and other countries with a high or very high HDI.

## Materials and methods

### Ethics

This study was performed in accordance with the Declaration of Helsinki and approved by the local ethics committee of the Faculty of Medicine of Ondokuz Mayıs University, Samsun, Türkiye (application number: 2024000476; acceptance date: 22/10/2024 and acceptance number: 2024/476). All patients provided written informed consent prior to participation in the study.

### Study design

This retrospective study included patients who were ≥18 years of age, had a histopathologically confirmed diagnosis of gynecologic cancer, and had presented to our radiation oncology department between January 2012 and December 2022. Additional inclusion criteria required that patients had been evaluated by the multidisciplinary gynecologic oncology tumor board and had undergone treatment and follow-up in our radiation oncology department. Patients were excluded if they did not meet the inclusion criteria or if they had a diagnosis of second cancer other than gynecologic cancer or if they had a diagnosis of inoperable corpus uteri cancer; all treatments and follow-up visits for these cases were conducted at other oncology centers due to the unavailability of a suitable BRA applicator.

### Patients’ evaluation, treatment and follow-up

Patients’ pre-treatment evaluations, treatments, post-treatment follow-ups, and diagnoses in case of recurrence were performed according to the then-current European Society for Radiotherapy and Oncology (ESTRO) and National Comprehensive Cancer Network (NCCN) guidelines specific to each disease site.

### Data collection

Data were collected regarding patient age, year of diagnosis, tumor site, histopathological diagnosis, and staging information based on the International Federation of Gynecology and Obstetrics (FIGO) system [in its current form of versions in NCCN guidelines for; endometrial carcinoma (2018), uterine sarcoma (2009), cervical cancer (2018), ovarian cancer (2017), vulvar cancer (2021) and vaginal cancer (2009)] and the Surveillance, Epidemiology, and End Results (SEER) summary staging classification (2018) by the United States National Cancer Institute, depending on the gynecologic cancer type. Treatment characteristics data were also recorded, including surgical status (operable or inoperable), RT purpose (curative, palliative, adjuvant, or definitive), RT technique (EBRT and/or BRA), along with information about relapse and survival status.

### Statistical analysis

Locoregional control, distant control, death from any cause or disease, disease-free survival (DFS), and overall survival (OS) were recorded and analyzed. DFS was defined as the time from diagnosis to first relapse at any site, whereas OS was defined as the time from diagnosis to death from any cause. Descriptive statistics were used to summarize patient characteristics. The Kaplan-Meier method was utilized for survival analysis. Statistical analyses were conducted with SPSS software (Version 22.0; SPSS Inc., Chicago, IL, USA). A p-value of < 0.05 was considered statistically significant. Given the descriptive nature of this study and its aim to characterize practice patterns rather than identify independent prognostic factors, no multivariable analyses were performed. Survival outcomes are presented descriptively to reflect real-world experience without adjustment for confounders.

## Results

**Figure 1:**
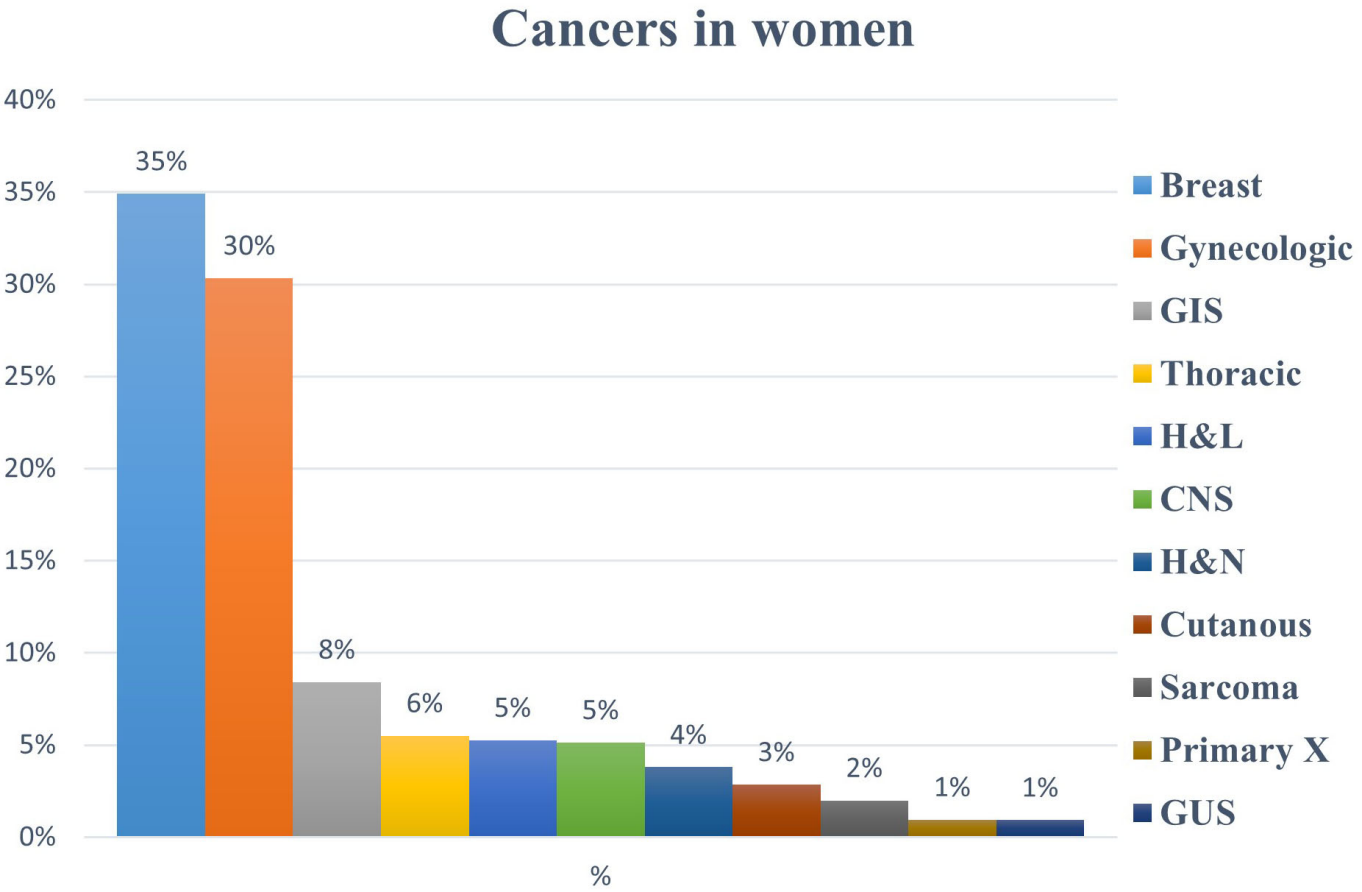
Percentage (%) distribution of all cancers in women treated in our radiation oncology practice; GIS: Gastrointestinal system, H&L: Hematology and lymphoma, CNS: Central nervous system, H&N: Head and neck, GUS: Genitourinary system.

**Figure 2:**
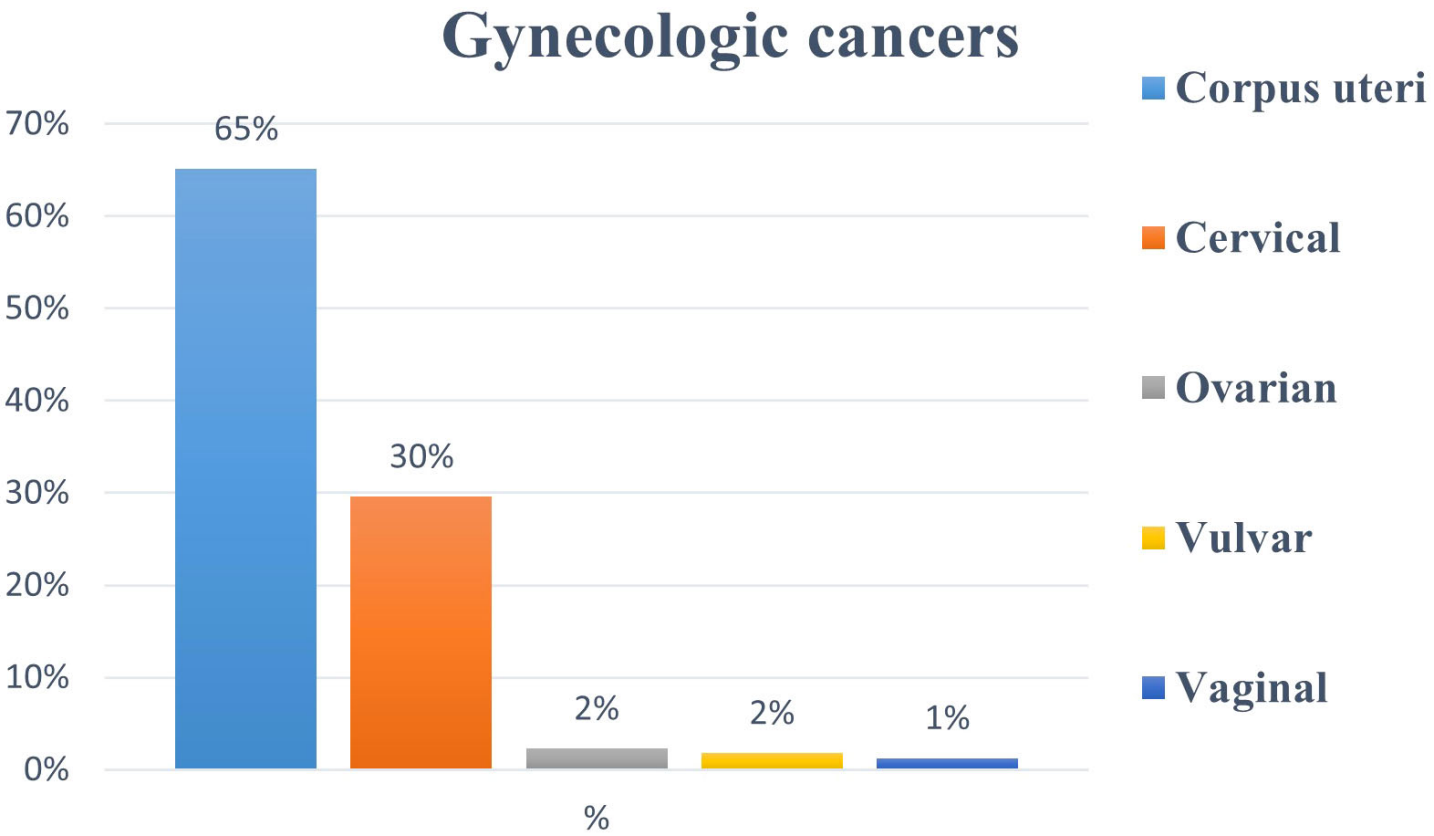
Percentage (%) distribution of all gynecologic cancers in our radiation oncology practice.

In total, 11,720 cancer patients were evaluated for RT over a 10-year period. Of these, 41% (n = 4,781) were women. Among female patients, gynecologic cancers comprised 30% of cases (n = 1,450), making them the second most common malignancy after breast cancer, which comprised 35% of cases (n = 1,669) (**Figure 1**). Among all cancers diagnosed in women (n = 4,781), corpus uteri cancer was the most common gynecologic malignancy, representing 20% (n = 944) of cases, followed by cervical cancer (9%, n = 429), ovarian cancer (<1%, n = 33), vulvar cancer (<1%, n = 26), and vaginal cancer (<1%, n = 18). When considering only gynecologic cancers (n = 1,450), these malignancies constituted 65%, 30%, 2%, 2%, and 1% of cases, respectively (**Figure 2**).

### Corpus uteri cancers

Among the 944 patients diagnosed with corpus uteri cancer, 98% (n = 927) presented after undergoing surgery. Of these, 575 patients underwent treatment and follow-up in our radiation oncology department. Among these patients, 99% (n = 572) received curative treatment: 97% (n = 552) had endometrial carcinoma and 3% (n = 20) had uterine sarcoma. The most common age group at diagnosis was 50–59 years, representing 36% (n = 211) of patients.

### Endometrial carcinoma

A median of 53 patients (range: 14–87, n = 554) with endometrial carcinoma underwent treatment and follow-up in our radiation oncology department annually. The most common histopathological subtype was endometrioid adenocarcinoma, observed in 85% (n = 469) of cases. The most frequently detected FIGO and SEER stages were stage I and localized stage, respectively, occurring in 69% (n = 384) of patients. Adjuvant RT was administered to 83% (n = 456) of curatively treated patients (n = 552). BRA alone was the most frequently performed RT technique, used in 51% (n = 235) of cases. Relapse occurred in 15% (n = 84) of curatively treated patients; 74% (n = 62) of these cases developed distant metastases (**Table 1**). Regardless of stage, the 2-, 5-, and 10-year DFS rates were 91.4%, 85.1%, and 83.6%, respectively; the 2-, 5-, and 10-year OS rates were 94.4%, 88.1%, and 82.0%, respectively. DFS and OS rates varied according to disease stage (**Table 2**).

**Table 1:** Characteristics of gynecologic cancer patients after initial diagnosis in a radiation oncology.

	Endometrial carcinoma (n = 554)	Uterine sarcoma (n = 21)	Cervical cancer (n = 269)	Ovarian cancer (n = 27)	Vulvar cancer (n = 26)	Vaginal cancer (n = 14)
	% (n)					
Age group						
< 30	0.2 (1)	–	2 (6)	4 (1)	–	–
30-39	2.9 (16)	5 (1)	10 (26)	–	–	–
40-49	10.1 (56)	33 (7)	25 (68)	19 (5)	4 (1)	7 (1)
50-59	35.6 (197)	43 (9)	32 (87)	44 (12)	19 (5)	22 (3)
60-69	36.8 (204)	9.5 (2)	20 (54)	22 (6)	23 (6)	57 (8)
≥ 70	14.4 (80)	9.5 (2)	11 (28)	11 (3)	54 (14)	14 (2)
FIGO stage						
I	69.3 (384)	57 (12)	24 (64)	–	46 (12)	21 (3)
II	14.8 (82)	19 (4)	40 (109)	–	23 (6)	29 (4)
III	15.5 (86)	19 (4)	31 (83)	7 (2)	19 (5)	36 (5)
IV	0.4 (2)	5 (1)	5 (13)	93 (25)	12 (3)	14 (2)
SEER stage						
Localized	69 (384)	57 (12)	24 (64)	–	46 (12)	22 (3)
Regional	30(168)	38 (8)	71 (192)	7 (2)	42 (11)	64 (9)
Distant	1 (2)	5 (1)	5 (13)	93 (25)	12 (3)	14 (2)
Treatment						
Curative	99.6 (552)	95 (20)	99 (267)	15 (4)	100 (26)	93 (13)
*- No RT*	17 (96)	40 (8)	2 (5)	–	8 (2)	–
-*Definitive RT*	–	–	62 (167)	100 (4)	46 (12)	62 (8)
-*Adjuvant RT*	83 (456)	60 (12)	36 (95)	–	46 (12)	38 (5)
Palliative RT	0.4 (2)	5 (1)	1 (2)	85 (23)	–	7 (1)
RT technique						
No	17 (96)	38 (8)	2 (5)	–	8 (2)	–
Yes	83 (458)	62 (13)	98 (264)	100 (27)	92 (24)	100 (14)
*-BRA alone*	51 (235)	–	–	–	–	–
*-EBRT alone*	13 (58)	69 (9)	9 (24)	100 (27)	100 (24)	29 (4)
*-EBRT + BRA*	36 (165)	31 (4)	91 (240)	–	–	71 (10)
Relapse^*^						
No	85 (468)	50 (10)	72 (192)	25 (1)	35 (9)	69 (9)
Yes	15 (84)	50 (10)	28 (75)	75 (3)	65 (17)	31 (4)
-*Locoregional*	26 (22)	30 (3)	36 (27)	67 (2)	65 (11)	75 (3)
-*Distant*	74 (62)	70 (7)	64 (48)	33 (1)	35 (6)	25 (1)

FIGO, International Federation of Gynecology and Obstetrics; SEER, Surveillance, Epidemiology, and End Results (National Cancer Institute, United States of America); RT, radiotherapy; BRA, brachytherapy; EBRT, external beam radiotherapy; *, only for patients treated curatively.

**Table 2:** Survival rates by stage in endometrial carcinoma and cervical cancer.

	Endometrial carcinoma	Cervical cancer
Disease-free survival		
FIGO stage I		
2-years (%)	96.3	87.5
5-years (%)	91.1	83.5
10-years (%)	91.1	81.4
FIGO stage II		
2-years (%)	87.8	84.8
5-years (%)	82.6	79.1
10-years (%)	78.6	70.5
FIGO stage III		
2-years (%)	73.0	65.5
5-years (%)	60.5	58.6
10-years (%)	55.5	54.8
p*	**0.000**	**0.002**
Overall survival		
FIGO stage I		
2-years (%)	99.2	93.8
5-years (%)	92.8	89.8
10-years (%)	87.7	85.3
FIGO stage II		
2-years (%)	89.0	88.9
5-years (%)	81.2	75.7
10-years (%)	71.6	69.2
FIGO stage III		
2-years (%)	90.7	83.5
5-years (%)	73.0	65.4
10-years (%)	67.2	60.2
p*	**0.000**	**0.039**
SEER localized stage		
2-years (%)	99.2	93.8
5-years (%)	92.8	89.8
10-years (%)	87.7	85.3
*SEER r*egional stage		
2-years (%)	89.9	88.9
5-years (%)	77.5	71.3
10-years (%)	69.1	65.4
p*	**0.000**	**0.000**

FIGO, International Federation of Gynecology and Obstetrics; SEER, Surveillance, Epidemiology, and End Results (National Cancer Institute, United States of America); * the p value refers to the comparison of the stages just above.

### Uterine sarcoma

A median of one patient (range: 0–5, n = 21) with uterine sarcoma underwent treatment and follow-up in our radiation oncology department annually. Leiomyosarcoma was the most common histopathological subtype, observed in 52% (n = 11) of cases. The most frequently diagnosed FIGO and SEER stages were stage I and localized stage, respectively, occurring in 57% (n = 12) of patients. Adjuvant RT was administered to 60% (n = 12) of curatively treated patients (n = 20). EBRT alone was the most frequently used RT technique, performed in 69% (n = 9) of cases. Relapse occurred in 50% (n = 10) of curatively treated patients; 70% (n = 7) of these cases developed distant metastases (**Table 1**). The 1-, 2-, and 5-year DFS rates were 84.2%, 66.5%, and 41.5%, respectively; the 1-, 2-, and 5-year OS rates were 95.0%, 80.0%, and 70.0%, respectively.

### Cervical cancer

A median of 25 patients (range: 4–49, n = 269) with cervical cancer underwent treatment and follow-up in our radiation oncology department annually. The most common age group at diagnosis was 50–59 years (32%; n = 87). Squamous cell carcinoma (SCC) was the predominant histopathological subtype, comprising 81% (n = 218) of cases. The most frequently diagnosed FIGO and SEER stages were stage II (40%; n = 109) and regional stage (71%; n = 192), respectively. Among patients treated with curative intent (n = 267), radical RT (EBRT with concurrent chemotherapy followed by BRA) was the primary treatment in 62% (n = 167) of cases. The most frequently used RT technique was EBRT combined with BRA, administered to 91% (n = 240) of patients. Relapse occurred in 28% (n = 75) of curatively treated patients; 64% (n = 48) of these cases developed distant metastases (**Table 1**). Regardless of stage, the 2-, 5-, and 10-year DFS rates were 79.4%, 73.9%, and 68.6%, respectively; OS rates at 2, 5, and 10 years were 88.6%, 76.3%, and 70.3%, respectively. DFS and OS rates varied according to stage (**Table 2**).

### Ovarian cancer

A median of three patients (range: 0–5, n = 27) with ovarian cancer underwent treatment and follow-up in our radiation oncology department annually. The most common age group at diagnosis was 50–59 years (44%; n = 12). Epithelial serous carcinoma was the predominant histopathological subtype, comprising 81% (n = 22) of cases. The most frequently diagnosed FIGO and SEER stages were stage IV and distant stage, respectively, with a prevalence of 93% (n = 25) each. In 85% (n = 23) of cases, RT was administered with palliative intent (**Table 1**). RT sites included the brain (33%; n = 9), lymph nodes (30%; n = 8), bone (22%; n = 6), vaginal cuff (11%; n = 3), and whole abdomen (4%; n = 1). All treatments were delivered via EBRT. The 1-, 2-, and 5-year DFS rates from the date of diagnosis were 85.2%, 51.9%, and 22.2%, respectively; the corresponding OS rates were 100.0%, 85.2%, and 29.6%, respectively. However, OS rates at 1, 2, and 5 years after RT were 37.0%, 22.2%, and 4.9%, respectively.

### Vulvar cancer

A median of three patients (range: 0–5, n = 26) with vulvar cancer underwent treatment and follow-up in our radiation oncology department annually. The most common age group at diagnosis was ≥ 70 years (54%; n = 14). SCC was the predominant histopathological subtype, observed in 96% (n = 25) of cases. The most frequently diagnosed FIGO and SEER stages were stage I and localized stage, respectively, with a prevalence of 46% (n = 12) each. Definitive RT was performed in 46% (n = 12) of cases, whereas adjuvant RT was required in 86% (n = 12) of patients who underwent primary surgical treatment (n = 14). All RT treatments (100%; n = 24) were delivered via EBRT. Relapse occurred in 65% (n = 17) of curatively treated patients; 65% (n = 11) of these cases developed locoregional disease (**Table 1**). The 1-, 2-, and 3-year DFS rates were 79.6%, 58.3%, and 49.4%, respectively; OS rates at 1, 2, and 3 years were 88.5%, 65.0%, and 52.0%, respectively.

### Vaginal cancer

A median of one patient (range: 0–4, n = 14) with vaginal cancer underwent treatment and follow-up in our radiation oncology department annually. The most common age group at diagnosis was 60–69 years (57%, n = 8). SCC was the predominant histopathological subtype, comprising 79% (n = 11) of cases. The most frequently observed FIGO and SEER stages at diagnosis were stage III (36%, n = 5) and regional-stage (64%, n = 9), respectively. RT was administered with curative intent in 93% (n = 13) of patients. The most frequently used RT technique was EBRT combined with BRA, performed in 71% (n = 10) of cases. Relapse occurred in 31% (n = 4) of curatively treated patients; 75% (n = 3) of these cases developed locoregional disease (**Table 1**). OS rates at 1 and 2 years were 64.0% and 57.0%, respectively.

### Palliative RT

A median of four patients (range: 0–9, n = 40) with gynecologic cancer underwent palliative treatment and follow-up in our radiation oncology department annually. The most common age group was 50–59 years (35%, n = 14). The ovary was the most frequent primary tumor site (57%, n = 23). The predominant histopathological subtypes were epithelial serous carcinoma in ovarian cancer (87%, n = 20), non-endometrioid carcinoma in uterine cancer (75%, n = 6), and SCC in cervical (75%, n = 3), vulvar (100%, n = 4), and vaginal cancers (100%, n = 1). At first diagnosis, the most commonly observed FIGO and SEER stages were stage III (70%, n = 28) and regional-stage disease (80%, n = 32), respectively. The primary indication for palliative RT was pain management (42%, n = 17), and the brain was the most frequently targeted site (30%, n = 12). All RT treatments were delivered using EBRT (**Table 3**). OS rates after diagnosis were 90.0% at 1 year, 67.5% at 2 years, and 20.0% at 5 years. After RT, the OS rates were 17.5% at 1 year and 10% at 2 years.

**Table 3:** Characteristics of patients receiving palliative radiotherapy for gynecologic cancers (n = 40).

	%	n
Age group		
< 30	2	1
30-39	–	–
40-49	12	5
50-59	35	14
60-69	28	11
≥ 70	23	9
Cancer site		
Corpus uteri	20	8
Cervix	10	4
Ovary	57	23
Vulva	10	4
Vagina	3	1
Time of metastasis		
At first diagnosis	15	6
At follow-up	85	34
Baseline FIGO stage		
I	5	2
II	10	4
III	70	28
IV	15	6
Baseline SEER stage		
Localized	5	2
Regional	80	32
Distant	15	6
Radiotherapy sites		
Brain	30	12
Bone	27	11
Lymph node	17	7
Vaginal cuff	13	5
Whole pelvic	10	4
Whole abdomen	3	1
Reasons for palliation		
Pain	42	17
Neurologic symptom	30	12
Bleeding	28	11

FIGO, International Federation of Gynecology and Obstetrics; SEER, Surveillance, Epidemiology, and End Results (National Cancer Institute, United States of America).

In total, 911 patients with gynecologic cancer underwent RT evaluation 922 times. RT was indicated in 88% (n = 811) of these evaluations. Among those receiving RT, the intent was curative in 95% (n = 771) of cases, and adjuvant RT was administered in 75% (n = 580). The distribution of RT techniques was as follows: EBRT combined with BRA in 53% (n = 419), BRA alone in 29% (n = 235), and EBRT alone in 18% (n = 146) (**Table 1**).

## Discussion

**Figure 3:**
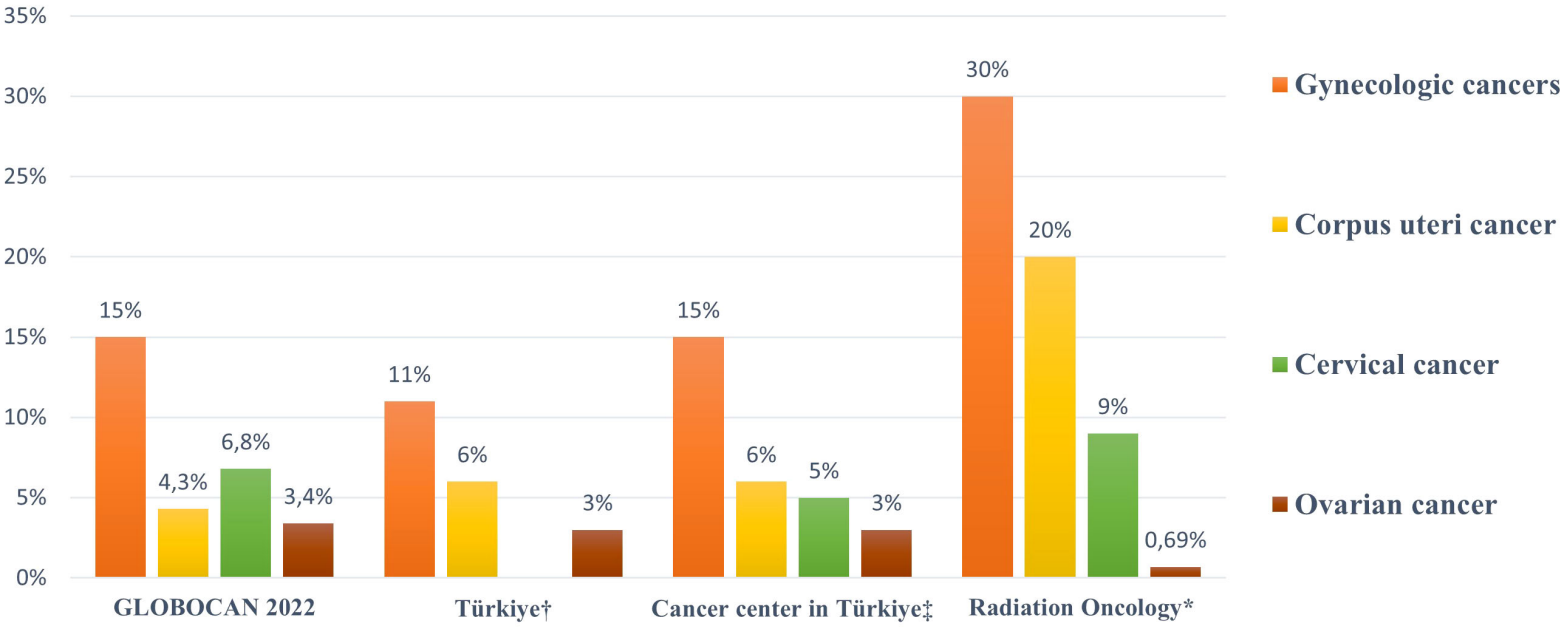
Comparison of the percentage (%) distribution of gynecologic cancers among all cancers in women between radiation oncology and other data sources; † Turkish Republic Ministry of Health [7]; ‡ Ege University Cancer Center [5]; * Our radiation oncology center.

After breast cancer, gynecologic cancers are the second most common group of malignancies in women: they constitute 11% of all cancers in women in Türkiye and 15% worldwide (2, 5). According to a study conducted by the Turkish Republic Ministry of Health, the three most prevalent gynecologic cancers among all female malignancies were corpus uteri cancer (ranked fourth overall), ovarian cancer (ranked sixth overall), and cervical cancer (ranked tenth overall) (7). The incidence of cervical cancer in Türkiye is particularly low; however, a comprehensive oncology center in Türkiye reported that cervical cancer was the second most common gynecologic malignancy after corpus uteri cancer (5). Their study also indicated that gynecologic cancers are the second most frequently diagnosed group of cancers in women after breast cancer (23%); corpus uteri cancer ranks fifth overall, cervical cancer ranks eighth, and ovarian cancer ranks ninth. According to GLOBOCAN 2022, the global ranking of gynecologic cancers differs, with cervical cancer ranking fourth overall, corpus uteri cancer ranking sixth, and ovarian cancer ranking eighth (8). In high or very high HDI countries, including Türkiye, corpus uteri cancer is the most frequently diagnosed gynecologic malignancy, whereas cervical cancer is predominant in low or medium HDI countries (9). In our radiation oncology practice, gynecologic cancers were the second most frequently treated malignancies after breast cancer, occurring 2.0–2.7 times more frequently compared with reports from other sources. The two most common gynecologic cancers were corpus uteri cancer (ranked second overall, occurring 3.3–4.6 times more frequently) and followed by cervical cancer (ranked third overall, occurring 1.3–1.8 times more frequently) (**Figure 3**). It should be noted that our cohort represents a selected referral population to a tertiary radiotherapy center and is not population-based. Therefore, direct comparisons with national or international registry data should be interpreted with caution, as differences may reflect referral patterns, clinical selection, and institutional expertise rather than true epidemiological variation.

The incidence of corpus uteri cancer has been increasing, particularly in high or very high HDI countries, including Türkiye. In these regions, as observed in our study, the most frequently diagnosed age group, FIGO stage, and SEER stage at presentation were 50–59 years, stage I, and localized stage, respectively (1, 4, 7). The standard first-line treatment for corpus uteri cancer is surgery; adjuvant therapy, including RT and/or chemotherapy, is administered based on disease stage and risk factors for recurrence (1, 10, 11). In our radiation oncology practice, 82% of operable patients with corpus uteri cancer underwent adjuvant RT, and 87% of those with endometrial carcinoma required BRA. Relapse rates for endometrial carcinoma range from 15% to 20% (12), whereas for uterine sarcoma are reportedly between 53% and 71% (13). In our study, relapse occurred in 15% of endometrial carcinoma cases and 50% of uterine sarcoma cases; distant metastases were the most common pattern of relapse for both, consistent with the literature. Studies from high or very high HDI countries (9) have revealed 5-year OS rates of up to 88.1% for endometrial carcinoma (14) and 74.0% for uterine sarcoma (15). In our study, the corresponding 5-year OS rates were 88.1% and 70.0%, respectively.

The incidence of cervical cancer has declined in developed countries (16). In regions with high or very high HDI, including Türkiye, the most commonly diagnosed age group, FIGO stage, and SEER stage at presentation were 40–49 years, stage I, and localized stage, respectively (4, 7, 17). However, in our radiation oncology practice, patients presented approximately one decade later and at more advanced stages compared with cohort analyses. For early-stage disease (FIGO IA–IB2), surgery is the standard first-line treatment; adjuvant therapy is administered based on stage and risk factors for recurrence. For advanced stages (FIGO IB3–IVA), radical RT remains the standard treatment (16). In our practice, most cervical cancer patients treated with curative intent were inoperable (62%) and received EBRT combined with BRA (91%). Relapse rates in cervical cancer ranges from 20% to 40% (18–20); in our study, relapse occurred in 28% of cases (most commonly as distant metastases), consistent with the literature. Studies have demonstrated 5-year OS rates for cervical cancer ranging from 56.8% to 76.5% in countries with high or very high HDI (9, 21). In our study, the 5-year OS rate was 76.3%.

Ovarian cancer is the most lethal gynecologic malignancy (4). In high- and very high-HDI countries, including Türkiye, the most commonly diagnosed age group, FIGO stage, and SEER stage at presentation were 50–59 years, stage III (26%), and distant stage (39–59%), respectively (4, 7, 22). Although our findings regarding age and SEER stage were consistent with these cohort analyses, we observed a higher frequency of FIGO stage IV disease, and that FIGO IV and SEER distant-stage cases occurred at substantially higher rates (26% vs. 93% and 59% vs. 93%, respectively). Surgery is the primary treatment for ovarian cancer, and chemotherapy serves as a key neoadjuvant and/or adjuvant therapy in the presence of adverse prognostic factors (23). RT is primarily used for palliative purposes in polymetastatic, symptomatic patients (85% in our study) and less frequently as a curative option with stereotactic irradiation techniques for oligorecurrent or oligometastatic disease (24). The relapse rate after first-line treatment is high, such that locoregional relapse occurs in up to 80% of cases (25, 26), consistent with findings in our curatively treated patients. Studies have shown 5-year OS rates for ovarian cancer ranging from 29.7% to 64.0% in high- and very high-HDI countries (9). In our study, the 5-year OS rate was 29.6%, which declined sixfold to 4.9% after RT. Unfortunately, studies indicate that survival outcomes are worse in ovarian cancer patients requiring RT (27).

Vulvar cancer comprises 5–8% of all gynecologic cancers, although it constituted only 2% of cases in our study. Its incidence has been increasing (28). In high- and very high-HDI countries, the most commonly diagnosed age group, FIGO stage, and SEER stage at presentation were ≥70 years, stage I, and localized stage, respectively (29, 30), consistent with our findings. Surgery is the standard first-line treatment; RT is administered as neoadjuvant or definitive therapy for inoperable patients, adjuvant therapy for high-risk cases, or palliative treatment for metastatic disease (31). In our radiation oncology practice, curative RT was delivered to 92% of vulvar cancer patients using EBRT alone, with half receiving definitive and the other half receiving adjuvant treatment. Relapse rates for vulvar cancer range from 22% to 61% (32, 33); in our study relapse occurred in 65% of cases (most commonly as locoregional disease), consistent with literature. The 2-year OS rate for vulvar cancer in high- and very high-HDI countries is reportedly between 47.2% and 72.0% (34, 35); the 2-year OS rate was 65.0% in our study.

Vaginal cancer is the rarest gynecologic malignancy (31). Primary vaginal cancer comprises approximately 10% of all vaginal cancers, corresponding to 1–2% of all gynecologic cancers (36). In high- and very high-HDI countries, it is most commonly diagnosed in individuals over 60 years of age (70%) and in FIGO stage I–II disease (67–74%) (31, 37). In our radiation oncology practice, although the frequency of primary vaginal cancer (1%) and age distribution (≥ 60 years, 71%) were consistent with these cohort analyses, a higher percentage of advanced FIGO stage disease (50%) was observed. Due to its rarity, management strategies for vaginal cancer are primarily based on a limited number of retrospective studies and extrapolated from cervical cancer treatment protocols. The standard first-line treatment for primary vaginal cancer is RT, typically involving EBRT with or without concurrent chemotherapy, followed by BRA. However, select early-stage cases may be treated with surgery or BRA alone (31, 37). In our practice, the majority of patients (93%) received curative treatment; 62% underwent definitive RT, most commonly EBRT followed by BRA (71%). Relapse rate for vaginal cancer is approximately 30% (38); in our study, relapse occurred in 31% of cases (most commonly as locoregional disease), consistent with literature. Studies have shown 2-year OS rates for vaginal cancer ranging from 62.0% to 91.0% in high- and very high-HDI countries (39), whereas the 2-year OS rate in our study was 57.0%. This lower survival rate can be attributed to the higher percentage of advanced-stage patients treated in our practice.

Palliative RT plays a crucial role in gynecologic cancer management, providing effective and well-tolerated symptom relief, particularly with modern RT techniques. Existing literature regarding palliative RT primarily focuses on specific tumor sites (40, 41), symptom control (42), and RT dose-fractionation schemas (43). However, studies evaluating palliative RT across all gynecologic cancers are extremely limited (44). Therefore, data concerning the most frequently treated primary tumor sites remain scarce. Reports have suggested that uterine corpus cancer is the most common indication for bleeding control, ovarian cancer for central nervous system symptom management, and vaginal cancer for general symptom relief (40, 42, 44). Patients requiring palliative RT frequently present at first diagnosis with advanced FIGO or SEER stage disease, as observed in our study, and RT achieves symptom control rates exceeding 70% (40–42, 44). Among patients undergoing palliative RT for gynecologic cancers, studies have demonstrated 1-year OS rates ranging from 21.0% to 28.0% in high- and very high-HDI countries (43, 44); in our study, the 1-year OS rate was 17.5%. This lower survival rate can be attributed to the high percentage (30%) of patients with brain metastases in our radiation oncology practice.

The main limitations of this study include its retrospective design, its single-center structure, the lack of analysis of potential technological changes during the study period, and the relatively small number of patients with ovarian, vulvar, and vaginal cancer. Survival estimates for ovarian, vulvar, and vaginal cancers due to the small number of cases, should be interpreted with caution, as they may be unstable and influenced by individual patient characteristics. Additionally, data regarding quality of life and treatment-related toxicities, which are critical factors when evaluating treatment outcomes, were unavailable.

## Conclusions

To our knowledge, this is the first study to comprehensively analyze the characteristics and survival outcomes of all gynecologic cancers in a radiation oncology practice. Our findings indicated that gynecologic cancers are the second most common malignancies among women in radiation oncology practice, after breast cancer. Corpus uteri and cervical cancers ranked higher in our radiation oncology center compared with reports from the International Agency for Research on Cancer, the National Cancer Registry Center, and comprehensive oncology center. Additionally, we observed that, except for ovarian cancer, most gynecologic cancers were referred for treatment at FIGO stages I–II. The most frequently performed RT technique was a combination of EBRT and BRA, with an overall BRA application rate of 82% in gynecologic radiation oncology. Furthermore, adjuvant RT was administered in 75% of cases. Given that our relapse and survival outcomes closely align with data from high- and very high-HDI countries, we believe that our findings broadly will reflect similar healthcare settings.

## Data Availability

The original contributions presented in the study are included in the article/supplementary material. Further inquiries can be directed to the corresponding author.

## Author’s note

The English in this document has been checked by at least two professional editors, both native speakers of English. For a certificate, please see: http://www.textcheck.com/certificate/tg7thQ.
